# Co-delivery of endometrial mesenchymal stem cells and macrophages with an electrospun patch suppresses endometrial fibrosis via IL-10 related signaling

**DOI:** 10.3389/fimmu.2026.1750456

**Published:** 2026-02-19

**Authors:** Jiangru An, Shuhong Li, Tianyi Ma, Yonghua Chen, J. Paul Santerre, Wenshuang Wang, Peng Ma, Xiaoqing Zhang

**Affiliations:** 1 International Joint Laboratory of Biomaterials and Tissue Regeneration, School of Basic Medicine, Binzhou Medical University, Yantai, Shandong, China; 2 Department of Obstetrics, Yuhuangding Hospital, Yantai, Shandong, China; 3 Institute of Biomedical Engineering, University of Toronto, Toronto, ON, Canada; 4 Li Ka Shing Faculty of Medicine, The University of Hong Kong, Pokfulam, Hong Kong SAR, China; 5 Department of Gynecology, Yuhuangding Hospital, Yantai, Shandong, China

**Keywords:** co-delivery, electrospun patch, endometrial fibrosis, human endometrial mesenchymal stem cells, immune-regulation, macrophages

## Abstract

**Introduction:**

Intrauterine adhesion (IUA) is characterized by endometrial fibrosis with partial or complete obliteration of the uterine cavity due to adhesion of the uterine wall. Currently, there is lack of effective strategies to address IUA and a strategy that can resolve endometrial fibrosis is needed. Human endometrial mesenchymal stem cells (H-EMSCs) and macrophages (mø) both reside in endometrial tissues and are important for endometrial repair. However, whether co-delivery of H-EMSCs and mø using a biocompatible biomaterial platform could address endometrial fibrosis and enhance repair remains unknown.

**Methods:**

This study developed a H-EMSCs-mø co-delivery system using an electrospun polycaprolactone-hyaluronic acid (PCL-HA) membrane and established a rat endometrial damage model. The effects of the co-delivery system on endometrial tissue fibrosis, M1 and M2 mø phenotypic marker modulation, and the pro-inflammatory (TNF-α) and anti-inflammatory (IL-10) factor production were investigated. The mechanisms involved in the interactions between H-EMSCs and mø were also delineated. All data were analyzed using analysis of variance with Tukey’s test for pair-wise comparisons or an independent samples t-test where appropriate.

**Results:**

In the rat endometrial damage model, H-EMSCs and mø co-delivery system (PCL-HA/H-E/mø) could significantly reduce endometrial fibrosis at day 14 after implantation vs. PCL-HA alone or H-EMSCs single-delivery (PCL-HA/H-E). In addition, PCL-HA/H-E/mø supported M1 (CD80, CD86) to M2-type mø phenotypic marker change and enhanced MSC marker (CD90) expression at days 3, 7 and 14, when compared to PCL-HA and PCL-HA/H-E groups. Moreover, PCL-HA/H-E/mø system had lower TNF-α gene expression but higher IL-10 gene and protein expression at day 7 post-implantation, suggesting an overall change from a more pro-inflammatory to a more wound-healing endometrial tissue microenvironment. In probing the mechanisms underlying the anti-fibrotic effect of the PCL-HA/H-E/mø system, it was found that IL-10 could have significantly reduced endometrial tissue fibrosis.

**Discussions:**

Co-delivery system reduced fibrosis and supported an M1-type to M2-type overall change of the mø phenotypes in the endometrial tissue damage model. IL-10 was one of the important factors in inhibiting endometrial fibrosis in the co-delivery system. This study provided significant insights into using a co-delivery system PCL-HA/H-E/mø, to effectively alleviate endometrial fibrosis and potentially for a more effective treatment of IUA.

## Graphical Abstract

**Figure:**
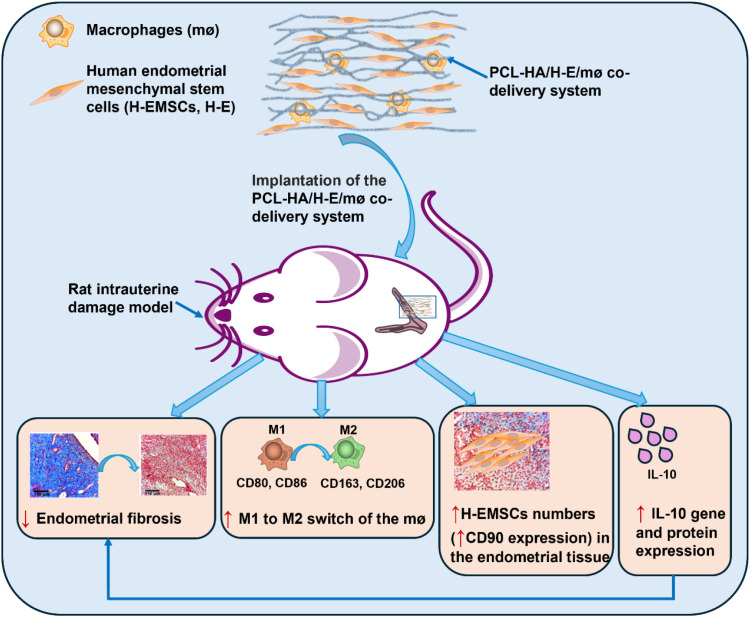

## 1 Background

Intrauterine adhesion (IUA), which is typically a uterine manifestation of Asherman’s syndrome, is characterized by endometrial fibrosis, with partial or complete obliteration of the uterine cavity due to adhesions of the uterine wall (1). IUA can be caused by trauma, infection and repeated curettage (2), and is a leading cause of uterine infertility worldwide (3). At present, the gold-standard clinical treatment for IUA is transcervical resection of adhesion (TCRA), in which an intrauterine device and balloon are inserted to expand the narrow uterine cavity (4). However, TCRA can have low effectiveness, and the reoccurrence rate post-surgery is up to 62.5% (5). The root cause for such a high reoccurrence rate is that TCRA relies on the endometrium’s self-repair capacity, instead of fundamentally addressing the underlying fibrotic pathology (6). IUA treatment would therefore benefit from a strategy to prevent fibrosis and facilitate the natural repair of the damaged endometrium.

Human endometrial mesenchymal stem cells (H-EMSCs) have self-renewal, immunomodulation capabilities and can promote new endometrial tissue formation and increase endometrial thickness after menstruation (7, 8). In other work, it has been found that monocyte-derived macrophages (mø) play a crucial role in endometrial repair and regeneration after injury (9). Mø are well-acknowledged to be key regulators of inflammation, fibrosis, tissue repair and regeneration (10). Specifically, mø can remove endometrial tissue debris through phagocytosis and produce a number of pro-inflammatory and anti-inflammatory cytokines, and growth factors to regulate the micro-environment, thereby contributing to the repair and regeneration of the endometrial tissues (11). Mø are classified into many differentiated phenotypes which fall under two major categories, each with their own multitude of sub-phenotypes: the classically activated mø sub-types (M1) and alternatively activated mø subtypes (M2) (12). Mø can also influence the survival, proliferation and migration of MSCs. Specifically, M2 mø can enhance bone marrow MSCs growth, proliferation and engraftment (13), while M1 mø can inhibit bone marrow MSCs proliferation and induce their apoptosis (14). On the other hand, MSCs can regulate mø via cell-cell contact or paracrine signaling mechanisms (15, 16). For example, the exosomes derived from umbilical-cord and placenta derived MSCs could induce the polarization of M1 pro-inflammatory mø towards the M2 anti-inflammatory phenotype mø, which enhanced the endometrial tissue repair (17, 18).

Direct injection of MSCs has been used for endometrial tissue repair (19, 20), but the benefit is limited due to a low survival rate and short retention time of the transplanted MSCs (21). Biomaterials could be designed as carriers for MSCs to address those issues associated with cell injection, as biomaterials can mimic the morphology of the natural extracellular matrix (ECM) and provide a proper microenvironment for cell attachment and proliferation (22). However, using biomaterials as a vehicle to deliver cells for treating endometrial tissue fibrosis and IUA has not been well explored. Polycaprolactone (PCL) is an organic polymer that has strong mechanical properties, good biocompatibility and is biodegradable over defined periods (23). Hyaluronic acid (HA) is an ECM-derived linear anionic polysaccharide, and has demonstrated excellent hydrophilicity, biocompatibility, biodegradability and non-immunoreactivity (24). In a previous study carried by our group, a PCL-HA electrospun membrane was fabricated to be morphologically similar to the native ECM, with ultrafine continuous fibers, high surface to volume ratio and high porosity (25). This PCL-HA scaffold supported H-EMSCs’ attachment, proliferation, enhanced H-EMSCs’ expression of wound-healing genes IL-10, VEGFA, TGF-β but suppressed their expression of tissue inflammation gene IL-6 (25). In addition, PCL-HA also supported MSC markers CD90 and Meflin expression of the seeded H-EMSCs (25). All of our previous findings have established PCL-HA as a model biomaterial platform for endometrial tissue repair and IUA treatment.

In this study, considering the fact that both H-EMSCs and mø play important roles in endogenous endometrial regeneration, we investigated the co-delivery of H-EMSCs and mø on PCL-HA membranes for treating endometrial fibrosis and IUA. The work also delineated some of the mechanisms involved in the interactions between H-EMSCs and mø when co-delivered with PCL-HA.

## 2 Methods

All chemicals of this work were purchased from Solarbio Life Sciences and used as is unless stated otherwise.

### 2.1 Isolation and culture of H-EMSCs

Human endometrial tissue pieces (released into menstrual blood) were obtained from the Yuhuangding Hospital of Yantai (ethics approval number: 2023-043). 15 ml PBS (P1020, Solarbio) containing 1% penicillin/streptomycin (PB180120, Procell) was added to 50 ml centrifuge tubes, and human endometrial tissue samples were collected in the tubes and transported to the processing lab on ice. The endometrial tissue was rinsed with PBS, cut into 1 mm³ pieces and digested with collagenase type I (1 mg/ml, BS163, Biosharp) for 60 minutes at 37°C in a constant temperature oscillator (80 r/min). DMEM/F12 complete medium (PM150312, Procell, containing 10% FBS (164210, Procell) and 1% penicillin/streptomycin) was used to terminate the digestion. The cells were centrifuged at 1000 r/min for 5 min and the cell pellet was re-suspended with DMEM/F12 medium and seeded into T75 TCPS culture flasks to obtain P1 (passage 1) H-EMSCs. Culture medium was changed 24h immediately after seeding and then changed every 2–3 days in culture. When H-EMSCs reached 80%-90% confluency, they were passaged using a ratio of 1:2. P4 (passage four) H-EMSCs were used for all the experiments in this study, in accordance with previous reported studies (26–28). The clinical information of patients who donated endometrial tissue samples of the study can be found in the **Supplementary Table 1** below. Characterization of the isolated H-EMSCs including (morphology, cell doubling time, flow cytometry for CD90, CD73 and CD45, colony forming capability, adipogenic, osteogenic and chondrogenic differentiation) was already performed in a previous study carried by our group (25), so it was not repeated in the current study.

### 2.2 Fabrication of PCL-HA electrospun membranes

PCL-HA electrospun membranes were fabricated according to our previously established methods (25). Specifically, PCL: HA (80:20) were added to 10 mL hexafluoro-isopropanol and the solution was magnetically stirred for 12h to obtain transparent spinning solutions. The solution was poured into a syringe and the electrospinner (Ne300, Inovenso Inc.) was set to have a flow rate of 1.5 mL/h, a spinning voltage of 12.6 kV and a spinning distance of 20 cm. After electrospinning, the PCL-HA electrospun membranes (2.5 cm×0.5 cm) were dried in a vacuum oven for 48h to remove residual solvent and then stored in a desiccator in dark until use. Characterization studies of the PCL-HA electrospun membranes include scanning electron microscopy, fiber diameter, material porosity % and degradation rate, as well as the material biocompatibility (the viability, proliferation and metabolic activities of the H-EMSCs) were already carried in our previous study (25), so those were not repeated in the current study.

### 2.3 H-EMSCs mono-delivery and H-EMSCs/Mø co-delivery via PCL-HA membrane

PCL-HA electrospun membranes were placed into tissue culture discs and sterilized with 70% ethanol (overnight) and then PBS was added to remove all residual ethanol. 1×10^6^ H-EMSCs suspended in 50 μl or 1×10^6^ H-EMSCs+0.25×10^6^ Mø [isolated from human peripheral blood based on our lab’s previous protocol (29, 30)] suspended in 50 μl were seeded onto the PCL-HA electrospun membranes for the H-EMSCs monoculture and H-EMSCs/Mø co-culture conditions, respectively. The cell seeding density and the H-EMSCs:Mø ratio in the co-culture condition were determined according to our group’s previously published studies (31, 32). The cell-seeded PCL-HA electrospun membranes were implanted into the rat endometrial damage model 24h post-cell seeding.

### 2.4 Establishing the rat endometrial damage model and implantation of PCL-HA patches

Female Sprague-Dawley (SD) rats (220–250 g, 8–10 weeks old) were purchased from Jinan Pengyue Biotechnology Co., Ltd. The rats were maintained at 22 °C with 12 h/12 h light and dark cycle and served with adequate food and water before the experiments. Ninety estrus female SD rats were divided into five groups: Sham operation group, natural repair group (NR), PCL-HA group, PCL-HA/H-E group and PCL-HA/H-E/mø group, with three time points (day 3, day 7, and day 14, 6 rats/time point) for each group. All rats were anesthetized by intraperitoneal injection of pentobarbital sodium (30 mg/kg), shaved in the middle and lower abdominal wall, and sprayed with 75% ethanol to disinfect the operation area. Under the conditions of aseptic operation, the rats’ abdominal wall was cut layer by layer, to fully expose the bilateral uterine horn, and a small incision was made near the fallopian tube of the uterus. A 16G syringe needle was inserted to scratch the uterine wall 8–10 times until the uterine surface became rough and bleeding, to establish the physical endometrial damage model. After flushing with PBS, the PCL-HA, PCL-HA/H-E and PCL-HA/H-E/mø patches were implanted to the surface of the damaged endometrium and it was confirmed that the patches were tightly adhered to the damaged endometrium, then the uterine wall incision was closed with 6–0 absorbable sutures (**Supplementary Figure 1**).

For the IL-10 inhibition experiments, 18 female SD rats in estrus were divided into the following three groups, with 6 rats in each group: PCL-HA/H-E group: PCL-HA electrospun membrane (1×10^6^ H-EMSCs) was transplanted; PCL-HA/H-E/mø group: PCL-HA electrospun membrane (1×10^6^ H-EMSCs + 0.25×10^6^ mø) was transplanted; PCL-HA/H-E/mø/IL-10 inhibitor group: PCL-HA electrospun membrane (1×10^6^ H-EMSCs and 0.25×10^6^ mø) was implanted, IL-10 inhibitor (Ossirene (HY-101019, MCE), 3 mg/kg) was injected into the rat uterine cavity at the time of implantation.

### 2.5 Collection of endometrial tissue samples and preparation of paraffin and frozen sections

At 3, 7 and 14 days, the rats from each group were anesthetized, and the endometrial tissue samples were collected. Specifically, a longitudinal incision was made along the uterine segment and then it was spread out flat. Then, the endometrial layer and the underlying muscularis were separated gently according to the junctions between the two. The endometrial tissue samples were washed with PBS and fixed with 4% paraformaldehyde (BL539A, Biosharp) at 4 °C for 48h. For the IL-10 inhibition experiment, the samples were taken at day 7. Subsequently, paraffin and frozen sections were prepared for the histology (Masson staining) and immunofluorescence staining experiments were carried out. For qRT-PCR experiments, the endometrial tissues were collected and stored in liquid nitrogen before use.

For the paraffin section preparation, the fixed endometrial tissues were cut into 3 mm-long segments, dehydrated and embedded with the following process: 75% ethanol (2h) →85% ethanol (2h) →95% ethanol (1h x2) →100% ethanol (1h x2) →xylene (30 min x2) →wax (1h) →embedding. The endometrial tissue samples were cut to a thickness of 5 μm using a microtome (Leica Biosystems Inc). Deparaffinization was achieved using the following procedure: xylene (15 min x2) →100% ethanol (10 min x2) →95% ethanol (5 min x2) →85% ethanol (5 min) →75% ethanol (5 min) →distilled water (1 min).

For the frozen section preparation, the fixed tissue samples were dehydrated in 30% sucrose for 48h. Then, tissue-Tek O.C.T. frozen section embedding medium (4583, Sakura Finetek Japan Co., Ltd.) was used to embed the endometrial tissue samples. Thermo Scientific™ CryoStar™ NX50 Cryostat was used to obtain frozen tissue sections with a 10 μm thickness.

### 2.6 Masson’s Trichrome staining

Masson’s Trichrome staining kit (G1340, Solarbio) was used to stain the endometrial tissue sections according to the manufacturer’s instructions. After staining with the dye solution, the tissue sections were rapidly dehydrated with 95% ethanol (3s) and 100% ethanol (10s x3), treated with xylene (1 min x3) and sealed with neutral balsam. Endometrial tissue sections were observed under inverted histology microscope (Zeiss). The quantification of the Masson’s Trichrome staining area was performed using the “Area” analysis tool in Image J software (Version 1.54).

### 2.7 Immunofluorescence

The frozen sections were soaked in PBS for 10 min to remove the tissue-Tek O.C.T. frozen section embedding medium. 10% goat serum (SL038, Solarbio) was used for blocking (room temperature, 1h) and the sections were treated with primary antibody solution at 4 °C for overnight. The next day, the tissue sections were incubated at room temperature for 1h and washed with PBS (5 min x3). The sections were then treated with secondary antibody solutions for 1h in the dark at room temperature. Cell nuclei were counter-stained with DAPI (10 μg/ml, C0065, Solarbio) (room temperature, 10 min in the dark) and anti-fade mountant (S2100, Solarbio) was added to all tissue sections before they were imaged with a Nikon upright fluorescence microscope. The following lists the primary and secondary antibodies used: rabbit polyclonal to α-SMA (1:200, YT5053, Immunoway); mouse monoclonal to CD80 (1:200, sc-376012, Santa Cruz Biotechnology); rabbit polyclonal to CD86 (1:200, YT7823, Immunoway); rabbit monoclonal to CD163 (1:200, AB182422, Abcam); rabbit polyclonal to CD206 (1:200, YT5640, Immunoway); mouse monoclonal to CD90 (1:200, 66766-1-lg, Proteintech); rabbit polyclonal to IL-10 (1:200, YT5138, Immunoway); AlexaFluor^®^ 568 goat anti-rabbit IgG (1:200, A-11011, Thermo scientific) and AlexaFluor^®^ 488 goat anti-mouse IgG (1:200, A28175, Thermo scientific). Immunofluorescence image analysis was performed using Image J software (Version 1.54) according to our previously established protocols (31, 32). It should be noted that the immunofluorescence image quantification is based on positive staining area (%) of the proteins, which does not distinguish cell number from protein expression level. However, in this study, the cell distribution was generally uniform in the tissue sections, so cell number should not significantly affect the relative protein expression levels in the different conditions.

### 2.8 qRT-PCR

mRNA was extracted from the cells or endometrial tissues using the Trizol (R401-01, Vazyme) method. mRNA quality and quantity were checked with NanoDrop™ 1000 Spectrophotometer (Thermo Scientific), and the samples were stored in -80 °C freezer. cDNA was synthesized using the reverse transcription kit (R233-01, Vazyme), according to the manufacturer’s instructions. The obtained cDNA samples were diluted 10 times with nuclease free water and used for qRT-PCR. Reaction mixtures containing 10 μl 2 x ChamQ SYBR qPCR Master Mix (Q311-02, Vazyme), 0.4 μl forward primer (10 μM), 0.4 μl reverse primer (10 μM), 0.7 μl cDNA sample, 8.5 μl ddH_2_O were prepared. The qPCR reaction was performed with Roche LightCycler™ Real-Time PCR Detection System, using the following protocol: Pre-incubation: 95 °C for 10 min, Amplification (40 cycles): 95 °C for 10 s, 60 °C for 30 s, Melting curve: 95 °C for 15 s, 60 °C for 1 min, 95 °C for 15 s. The data was analyzed using the comparative 2^-ΔΔCt^ method. The forward and reverse primer sequences of the genes can be found in the following **Table 1**.

**Table 1:** The forward and reverse primer sequences of the genes (rat-α-SMA, rat-CD90, rat-TNF-α, rat-IL-10).

Gene Name	Primer Sequences
rat-α-SMA	Forward (5’–3’): CGTGACTACTGCTGAGCGTGA Reverse (5’–3’): TGCCCATCAGGCAGTTCGTAG
rat-CD90	Forward (5’–3’): CACTCTCCTGCTTTCAGTCTTGC Reverse (5’–3’): GGCTGAACTCATGCTGGATGG
rat-TNF-α	Forward (5’–3’): CCCAAATGGGCTCCCTCTCAT Reverse (5’–3’): TTGGTGGTTTGCTACGACGTG
rat-IL-10	Forward (5’–3’): CAGACCCACATGCTCCGAGA Reverse (5’–3’): CAAGGCTTGGCAACCCAAGTA

For the IL-10 inhibition experiment, the cells included the following groups: Control group: 5×10^4^ RFs (CRL-1764, ATCC) treated with DMEM/F12 complete medium; PCL-HA/H-E group: 5×10^4^ RFs treated with cell culture supernatant of H-EMSCs seeded on PCL-HA; PCL-HA/H-E/mø group: 5×10^4^ RFs treated with cell culture supernatant of H-EMSCs/macrophages co-seeded on PCL-HA; PCL-HA/H-E/mø/IL-10 inhibitor group: 5×10^4^ RFs treated with cell culture supernatant of H-EMSCs/macrophages co-seeded on PCL-HA and IL-10 inhibitor Ossirene (1μg/mL, according to manufacturer’s instructions). The cells were treated for 24h before mRNA extraction.

### 2.9 ELISA

A longitudinal incision was made along the uterine segment and then it was spread out flat. Then, the endometrial layer and the underlying muscularis were separated gently according to the junctions between the two. About 0.5g of endometrial tissue samples were collected and washed with pre-cooled PBS to remove blood, minced with ophthalmic scissors, homogenized using a tissue homogenizer and then the tissue samples were transferred to a 1.5ml centrifuge tube containing 200μl PBS, and centrifuged at 11200 rpm for 15 min at 4°C and stored on ice. The protein concentrations of the samples were measured using the BCA kit (PC0020, Solarbio) according to the manufacturer’s instructions. The absorbance was measured at 562 nm wavelength using a spectrometer (SpectraMax M2, Molecular Devices, LLC), and the protein concentrations of the samples were determined and recorded.

For the IL-10 quantification of the endometrial tissue homogenates, Rat IL-10 ELISA Kit (EK310/2, Lianke) was used according to the manufacturer’s instructions. Specifically, 100 μl diluted standard or sample solution was added to each test well and 100 μl sample diluents were added to the blank wells. Then, 50 μl of the detection antibody solution was added to each well and the plate was sealed and incubated at room temperature for 2h. After incubation, the plate was washed with 300 μl wash buffer/well for 6 times, and 100 μl of streptavidin-HRP was added to each well and incubated for 45 min. Finally, the plate was washed with 300 μl wash buffer/well for 6 times, and 100 μl of HRP substrate (TMB) was added to each well and incubated at room temperature in the dark for 15 min. 100 μl of stop solution was added to each well to terminate the reaction, and the absorbance was taken at 450 nm wavelength using SpectraMax M2 immediately.

### 2.10 Data analysis

All data were analyzed using SPSS 22.0 software (SPSS Inc., Chicago, IL) by analysis of variance (ANOVA) with Tukey’s test for pair-wise comparisons or an independent samples t-test, where appropriate. All experiments were repeated at least three times with at least three samples each time (N = 3, n=3) unless stated otherwise. Data are represented as mean ± S.E.M, *p* < 0.05 indicated statistical significance.

## 3 Results

This study established a rat endometrial damage model and probed the effects of a PCL-HA electrospun membrane-based co-delivery system containing H-EMSCs and mø, on resolving endometrial tissue fibrosis and promoting endometrial tissue repair. In addition, the regulation of the co-delivery system (PCL-HA/H-E/mø) on M1/M2 mø marker-expression shifts, the MSC marker expression as well as the generation of the proinflammatory and anti-inflammatory factors within the local endometrial tissue micro-environment was investigated. Finally, a prominent signaling pathway underlying the significant reduction of endometrial fibrosis by the PCL-HA/H-E/mø co-delivery system was discovered.

### 3.1 PCL-HA/H-E/mø co-delivery system reduced endometrial tissue fibrosis

**Figure 1:**
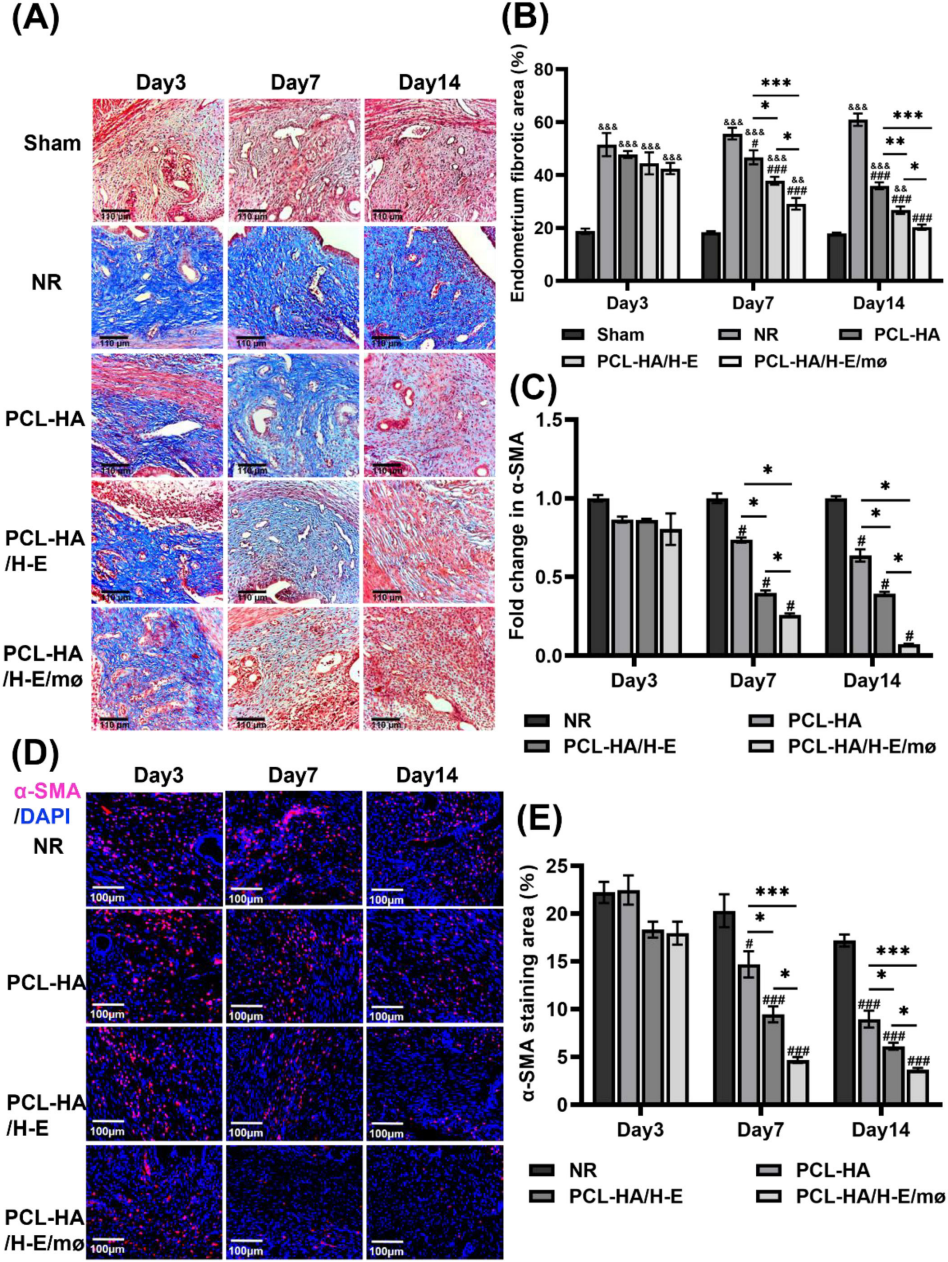
Masson staining of Sham, normal repair (NR), PCL-HA membrane alone, PCL-HA membrane with H-EMSCs mono-delivery (PCL-HA/H-E), PCL-HA membrane with H-EMSCs and mø co-delivery (PCL-HA/H-E/mø) groups, at day 3, 7 and 14. α-SMA gene and protein expression of the rat endometrial tissues in the NR, PCL-HA, PCL-HA/H-E, PCL-HA/H-E/mø groups, at day 3, 7 and 14. **(A)** Representative Masson images of the rat uterine tissues. Scale bar=110 µm. **(B)** Quantification of the endometrium fibrotic area (%). *p < 0.05, **p < 0.01, ***p < 0.001. #*p* < 0.05, ###*p* < 0.001 vs. NR. &&*p* < 0.01, &&&*p* < 0.001 vs. Sham. **(C)** α-SMA gene expression. *p < 0.001. #*p* < 0.001 vs. NR. **(D)** Representative images of α-SMA immunostaining of the uterine tissues. The nuclei were stained in blue while α-SMA was stained in red. Scale bar=;100 µm. **(E)** Quantification of the α-SMA immunostaining area (%). *p < 0.05, ***p < 0.001. #*p* < 0.05, ###*p* < 0.001 vs. NR.

As can be seen in **Figures 1A, B**, comparing the NR (damaged group without any treatment, stands for normal repair) condition with Sham, NR showed significantly higher fibrosis (blue area). Therefore, the endometrial damage animal model was validated. In addition, the implantation of H-EMSCs and mø co-delivery system (PCL-HA/H-E/mø) into the endometrial damage model showed greater effectiveness in reducing endometrium tissue fibrosis at day 7 vs. Sham, NR, PCL-HA alone or H-EMSCs mono-delivery system (PCL-HA, PCL-HA/H-E) (**Figures 1A, B**). At day 14, after implantation, the PCL-HA/H-E/mø repairing group had the smallest endometrial tissue fibrotic area vs. NR, PCL-HA, and the PCL-HA/H-E group (**Figures 1A, B**). Importantly, it was found that the endometrium fibrotic area of the PCL-HA/H-E/mø group was reduced to a level that was similar to the Sham group at day 14 (**Figures 1A, B**).

Since α-SMA is a well-acknowledged marker for endometrial tissue fibrosis (33, 34), the effects of PCL-HA/H-E/mø co-delivery system on the α-SMA gene and protein expression in the endometrium were also evaluated. It was found that the PCL-HA/H-E/mø treatment group had lower α-SMA gene expression in the endometrial damage tissue vs. the NR, PCL-HA, PCL-HA/H-E groups (**Figure 1C**), consistent with reduced tissue fibrosis (**Figures 1A, B**). The endometrial damage tissue α-SMA protein expression profile also matched well with the α-SMA gene expression data, indicating lower endometrial tissue fibrosis in the PCL-HA/H-E/mø condition (**Figures 1D, E**). Also, since the endometrium and the underlying muscularis were separated in the tissue processing procedures, the observed α-SMA gene and protein expression can be confirmed to be associated with the endometrial tissue, but not the muscularis.

### 3.2 PCL-HA/H-E/mø co-delivery system regulated M1/M2 mø marker-expression shifts

**Figure 2:**
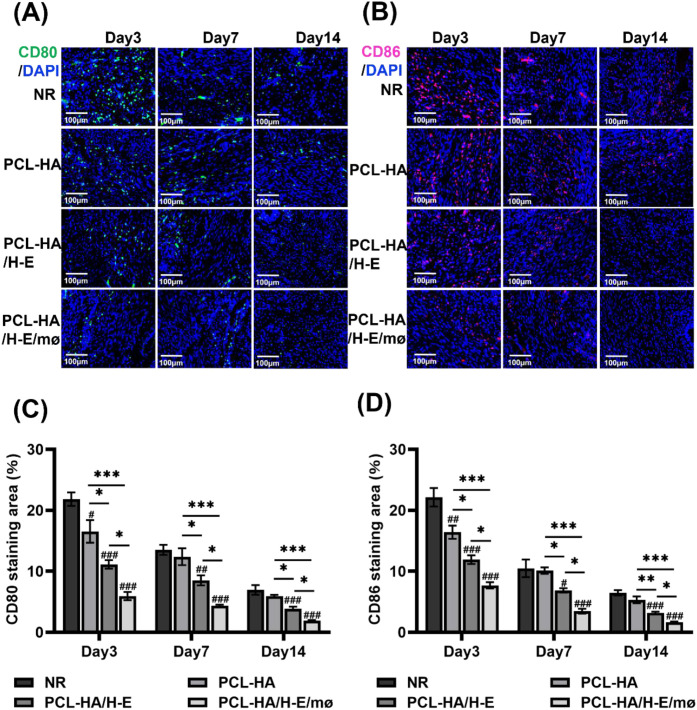
CD80 and CD86 immunostaining of rat uterine tissues of the NR, PCL-HA, PCL-HA/H-E and PCL-HA/H-E/mø groups at day 3, 7 and 14. **(A)** Representative images of CD80 immunostaining of the uterine tissues. The nuclei were stained in blue while CD80 was stained in green. Scale bar=100 µm. **(B)** Representative images of CD86 immunostaining of the uterine tissues. The nuclei were stained in blue while CD86 was stained in red. Scale bar=100 µm. **(C)** Quantification of the CD80 immunostaining area (%). *p < 0.05, ***p < 0.001. #*p* < 0.05, ##*p* < 0.01, ###*p* < 0.001 vs. NR. **(D)** Quantification of the CD86 immunostaining area (%). *p < 0.05, **p < 0.01, ***p < 0.001. #*p* < 0.05, ##*p* < 0.01, ###*p* < 0.001 vs. NR.

**Figure 3:**
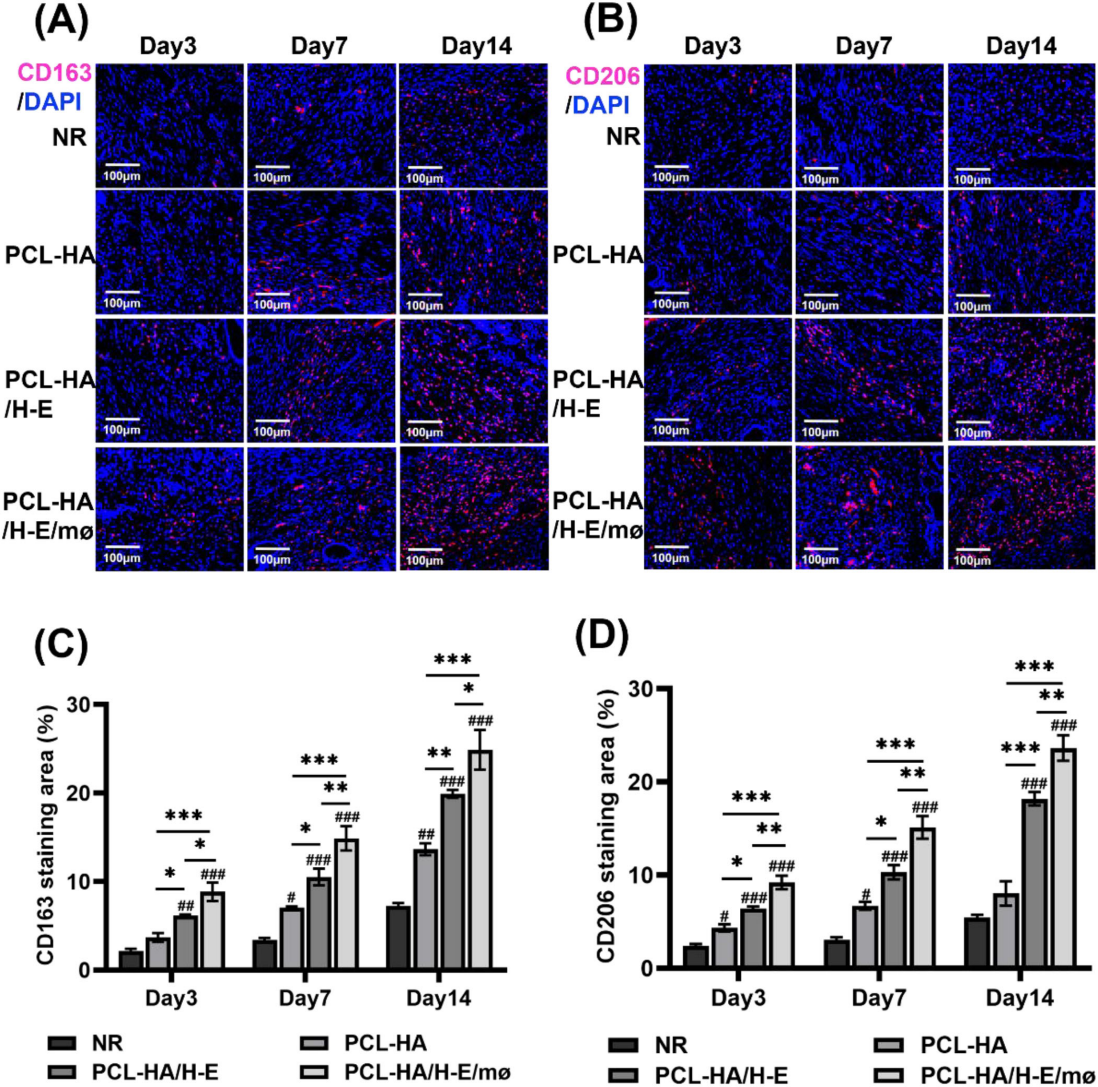
CD163 and CD206 immunostaining of rat uterine tissues of the NR, PCL-HA, PCL-HA/H-E and PCL-HA/H-E/mø groups at day 3, 7 and 14. **(A)** Representative images of CD163 immunostaining of the uterine tissues. The nuclei were stained in blue while CD163 was stained in red. Scale bar=100 µm. **(B)** Representative images of CD206 immunostaining of the uterine tissues. The nuclei were stained in blue while CD206 was stained in red. Scale bar=100 µm. **(C)** Quantification of the CD163 immunostaining area (%). *p < 0.05, **p < 0.01, ***p < 0.001. #*p* < 0.05, ##*p* < 0.01, ###*p* < 0.001 vs. NR. **(D)** Quantification of the CD206 immunostaining area (%). *p < 0.05, **p < 0.01, ***p < 0.001. #*p* < 0.05, ###*p* < 0.001 vs. NR.

Previous studies have shown that M1-type mø can enhance the inflammatory tissue microenvironment and can initiate tissue fibrosis, while M2 mø phenotypes are typically associated with tissue repair and wound-healing (35). Since the PCL-HA/H-E/mø treatment group significantly reduced fibrosis in the endometrial damage model, markers of the M1 (CD80, CD86) and M2 (CD163, CD206) mø were investigated in the different treatment groups (**Figures 2**, **3**). It was found that the expressions of M1-type (CD80, CD86) mø markers in the endometrial tissues were significantly lower in the PCL-HA/H-E/mø group vs. the NR, PCL-HA, PCL-HA/H-E groups at day 3, 7 and 14 time points (**Figure 2**). However, it was observed that the expression levels of M2-type (CD163, CD206) mø markers, in the endometrial tissues, were the opposite trend to the M1-type marker expression (**Figure 3**). Both CD163 and CD206 expression in the endometrial tissues were the highest for the PCL-HA/H-E/mø group at days 3, 7 and 14 when compared to the NR, PCL-HA and PCL-HA/H-E treatment groups (**Figure 3**).

### 3.3 PCL-HA/H-E/mø co-delivery system regulated CD90, IL-10 and TNF-α expression within the endometrial tissues

**Figure 4:**
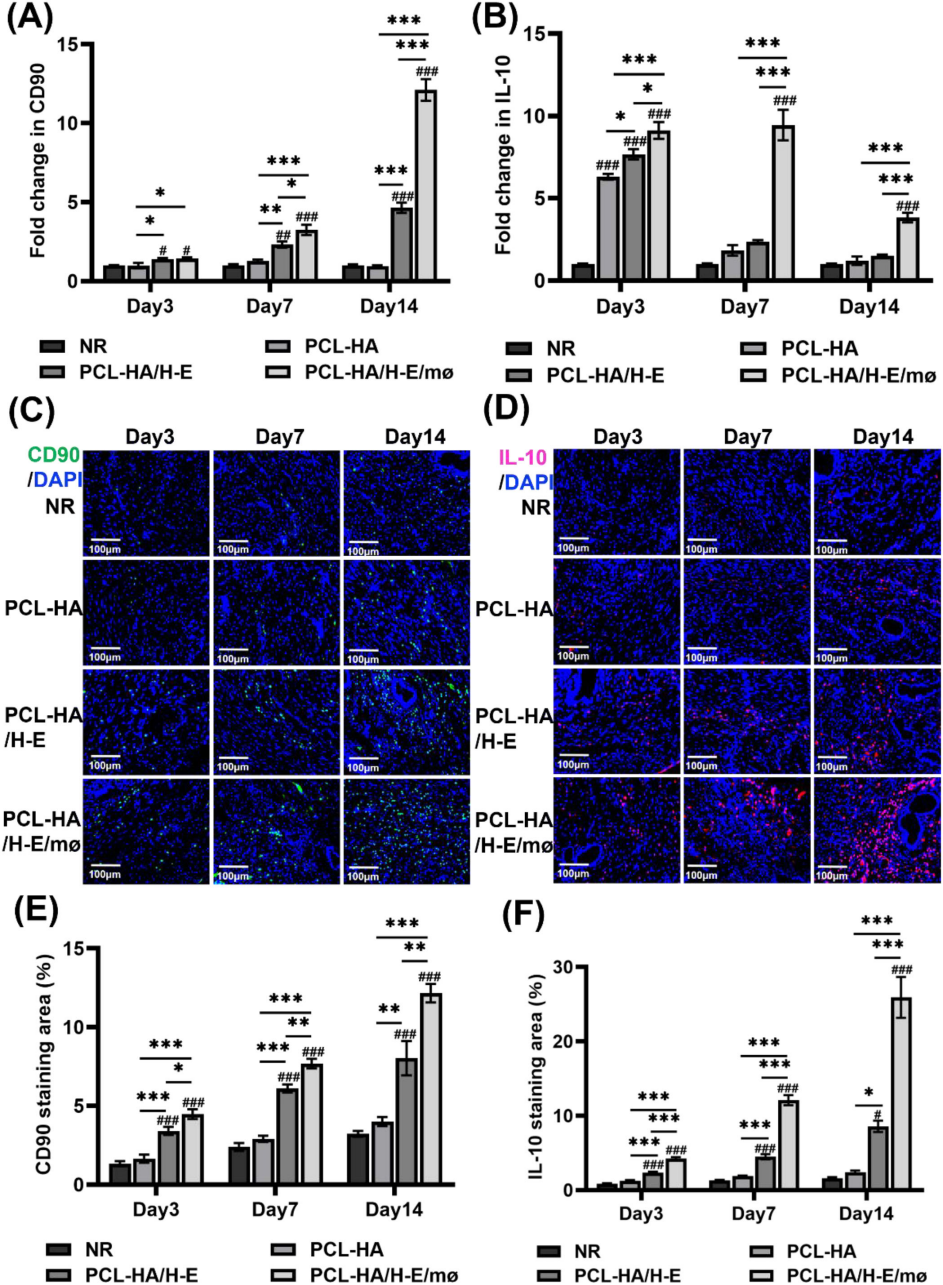
CD90, IL-10 gene expression and CD90, IL-10 immunostaining of rat uterine tissues of the NR, PCL-HA, PCL-HA/H-E and PCL-HA/H-E/mø groups at day 3, 7 and 14. **(A)** CD90 gene expression of the rat endometrial tissues. *p < 0.05, **p < 0.01, ***p < 0.001. #*p* < 0.05, ##*p* < 0.01, ###*p* < 0.001 vs. NR. **(B)** IL-10 gene expression of the rat endometrial tissues. *p < 0.05, ***p < 0.001. #*p* < 0.05, ##*p* < 0.01, ###*p* < 0.001vs. NR. **(C)** Representative images of CD90 immunostaining of the uterine tissues. The nuclei were stained in blue while CD90 was stained in green. Scale bar=100 µm. **(D)** Representative images of IL-10 immunostaining of the uterine tissues. The nuclei were stained in blue while IL-10 was stained in red. Scale bar=100 µm. **(E)** Quantification of the CD90 immunostaining area (%). *p < 0.05, **p < 0.01, ***p < 0.001. ###*p* < 0.001vs. NR. **(F)** Quantification of the IL-10 immunostaining area (%). *p < 0.05, ***p < 0.001. #*p* < 0.05, ###*p* < 0.001 vs. NR.

**Figure 5:**
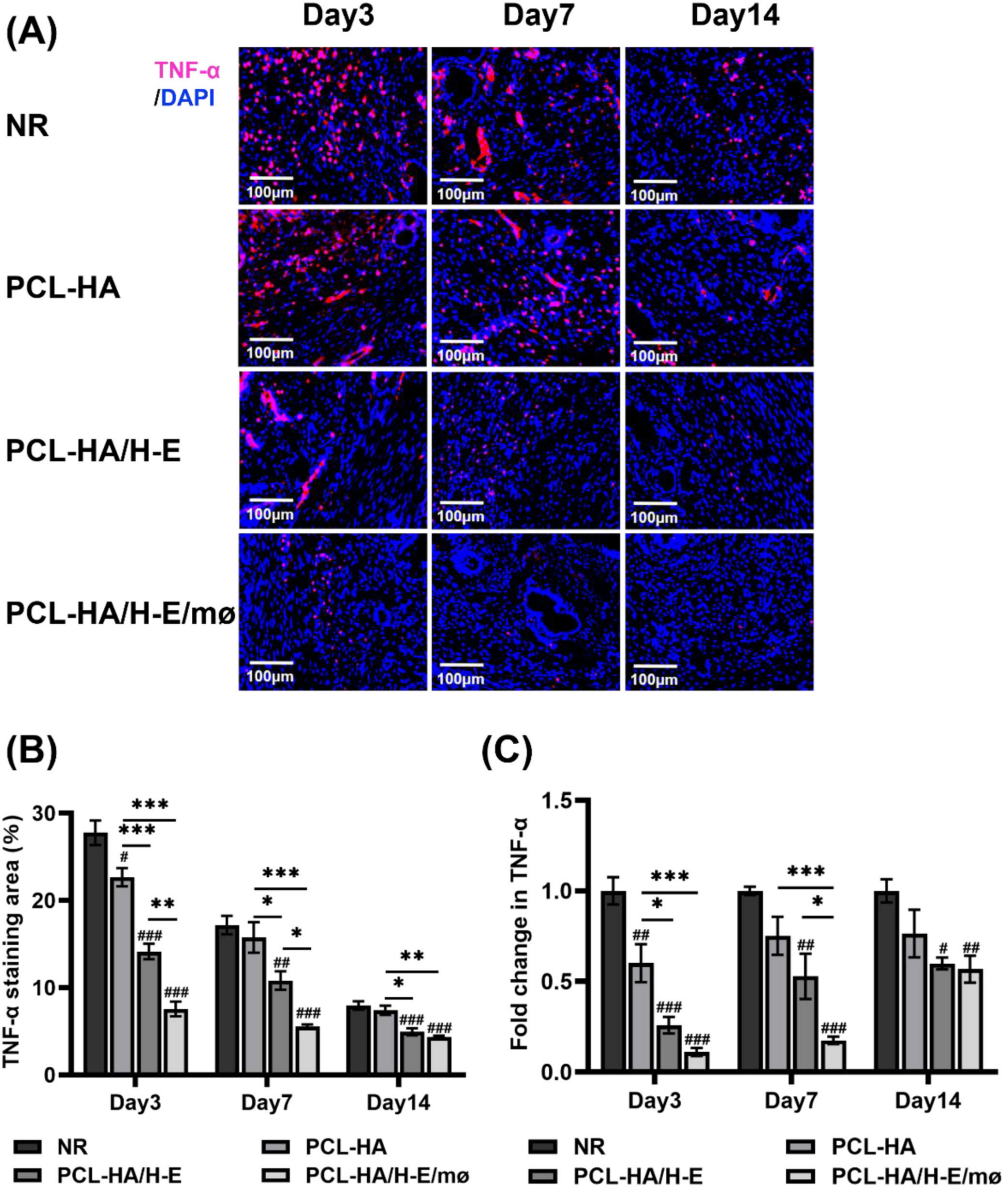
TNF-α immunostaining and TNF-α gene expression of rat uterine tissues of the NR, PCL-HA, PCL-HA/H-E and PCL-HA/H-E/mø groups at day 3, 7 and 14. **(A)** Representative images of TNF-α immunostaining of the uterine tissues. The nuclei were stained in blue while TNF-α was stained in red. Scale bar=100 µm. **(B)** Quantification of the TNF-α immunostaining area (%).*p < 0.05, **p < 0.01, ***p < 0.001. #*p* < 0.05, ##*p* < 0.01, ###*p* < 0.001 vs. NR. **(C)** TNF-α gene expression of the rat endometrial tissues. *p < 0.05, ***p < 0.001. #*p* < 0.05, ##*p* < 0.01, ###*p* < 0.001vs. NR.

The endometrial mesenchymal stem cell marker CD90, anti-inflammatory marker IL-10, as well as proinflammatory marker TNF-α expressions within the endometrial tissues of the different treatment groups, were assessed at days 3, 7 and 14 (**Figures 4**, **5**). It was observed that the mesenchymal stem cell marker CD90 gene expression was higher in the endometrial tissue that was implanted with PCL-HA/H-E/mø when compared with the PCL-HA/H-E, PCL-HA and NR groups at days 7 and 14 (**Figure 4A**). Similarly, the PCL-HA/H-E/mø group showed higher CD90 protein expression for immunostaining data, within the endometrial tissues vs. NR, PCL-HA, PCL-HA/H-E treatment groups at days 3, 7 and 14 (**Figures 4C, E**). The results of **Figures 4A, C, E** suggested that the implantation of PCL-HA/H-E/mø co-delivery system appeared to be enhancing H-EMSCs proliferation or was effectively recruiting more H-EMSCs from the local tissues during the repairing process of the damaged endometrial tissues.

When investigating IL-10 and TNF-α expressions within the endometrial tissues of the different treatment groups (**Figures 4B, D, F**, **5**), it was found that the co-delivery treatment group showed higher IL-10 gene expression level vs. NR, PCL-HA, PCL-HA/H-E at all time points (day 3, 7 and 14) (**Figure 4B**). Additionally, the IL-10 protein expression quantification matched the gene expression data, showing that the co-delivery group significantly upregulated the IL-10 protein expression in the endometrial tissue vs. all other treatment groups at days 3, 7 and 14 (**Figures 4D, F**). On the other hand, the gene and protein expression pattern of IL-10 for the different treatment groups displayed the opposite trends to that of TNF-α. Specifically, it was demonstrated that the PCL-HA/H-E/mø group had lower TNF-α gene (day 7 post-implantation) and protein expression (day 3, 7 post-implantation) vs. NR, PCL-HA, PCL-HA/H-E (**Figure 5**). At day 3, the co-delivery group showed lower TNF-α gene expression vs. NR and PCL-HA, however, no significant differences were detected between mono-delivery and co-delivery (**Figure 5C**).

### 3.4 IL-10 may inhibit α-SMA expression and endometrial tissue fibrosis

**Figure 6:**
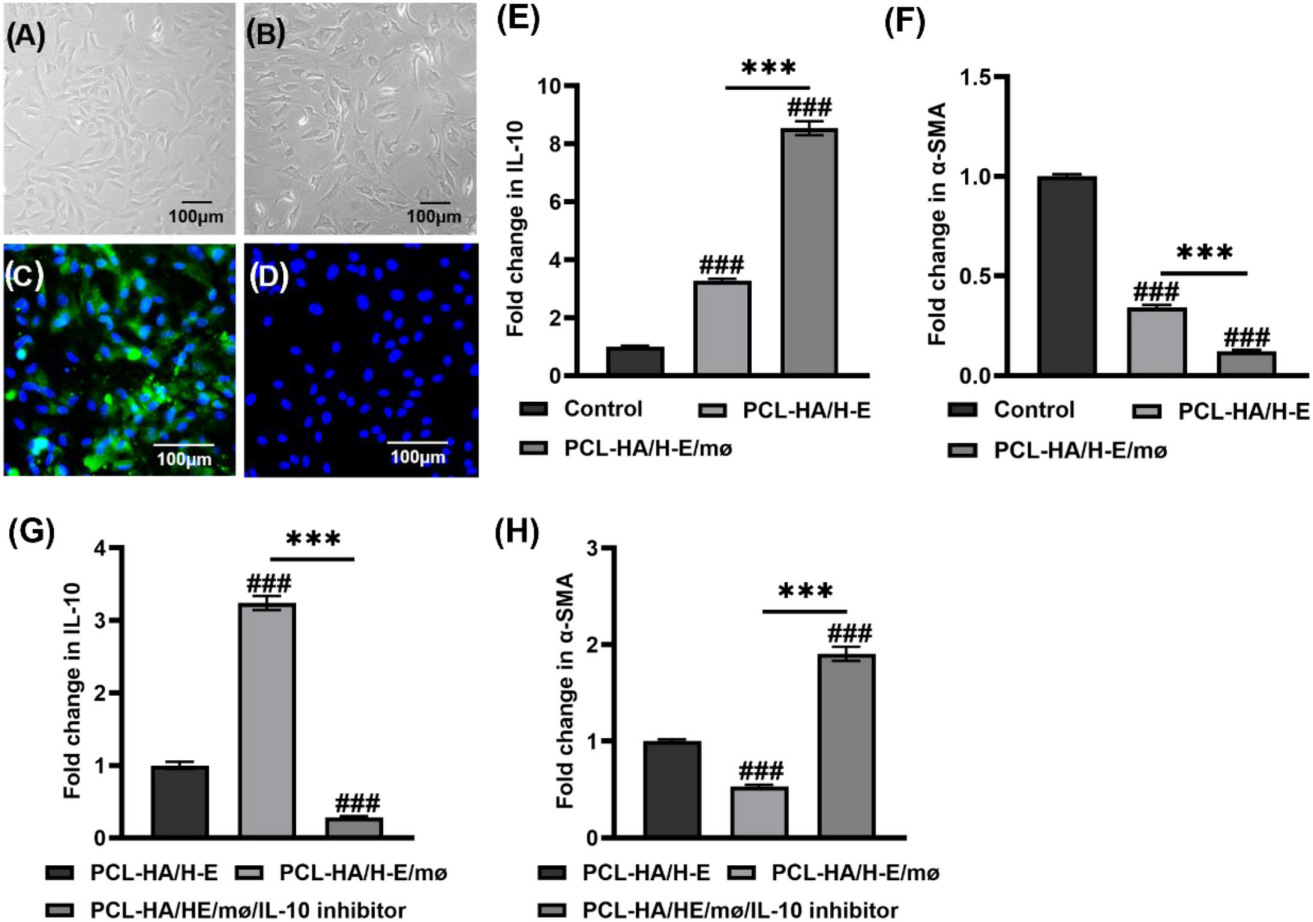
Assessment of **(A, B)** Representative images of rat RFs and immunostaining of **(C)** Vimentin (+staining) and **(D)** CD31 (-staining). Scale bar =100 µm. **(E)** IL-10 and **(F)** α-SMA expression of the RFs treated without (control) and with cell culture supernatant collected from PCL-HA/H-E or PCL-HA/H-E/mø. ***p < 0.001. ###*p* < 0.001 vs. Control. **(G)** IL-10 and **(H)** α-SMA expression of the RFs treated with cell culture supernatant collected from PCL-HA/H-E, PCL-HA/H-E/mø and PCL-HA/H-E/mø with IL-10 inhibitor (PCL-HA/H-E/mø/IL-10 inhibitor). ***p < 0.001. ###*p* < 0.001 vs. PCL-HA/H-E group.

**Figure 7:**
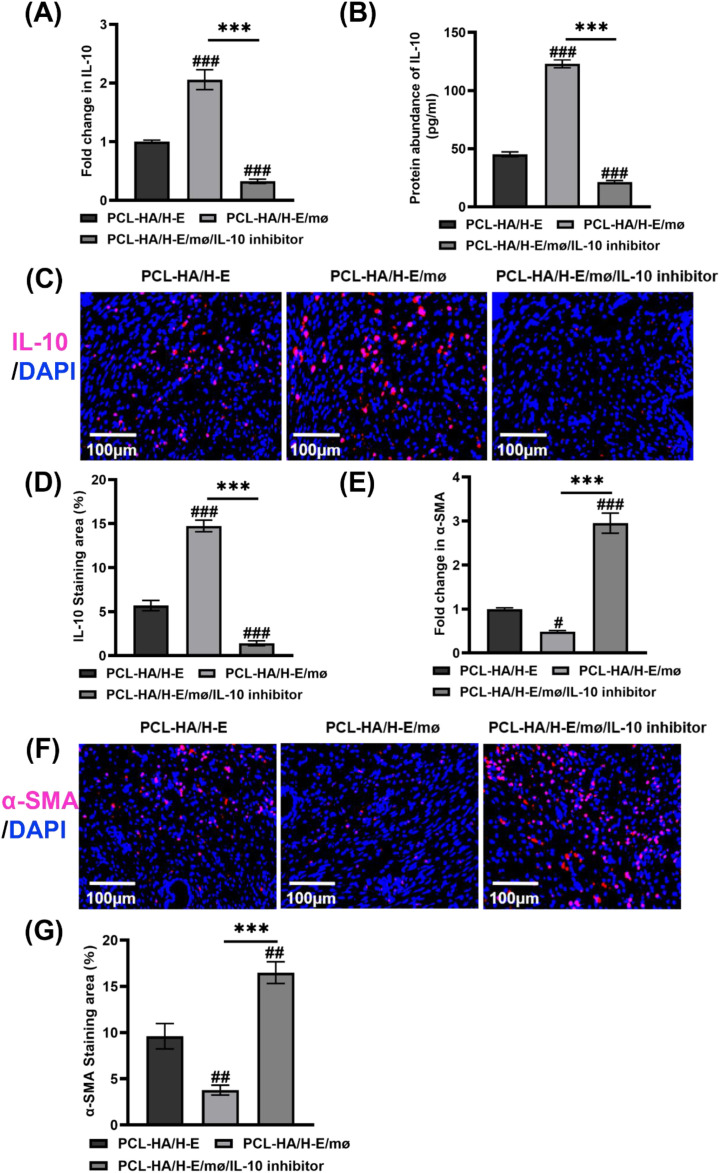
IL-10, α-SMA gene and protein expression of rat uterine tissues of the PCL-HA/H-E, PCL-HA/H-E/mø and PCL-HA/H-E/mø/IL-10 inhibitor groups at day 7. **(A)** IL-10 gene expression. **(B)** Quantification of IL-10 protein concentration (pg/ml). **(C)** Representative images of IL-10 immunostaining. Cell nuclei stained in blue while IL-10 stained in red. Scale bar=100 µm. **(D)** Quantification of IL-10 staining area (%). **(E)** α-SMA gene expression. **(F)** Representative images of α-SMA immunostaining. Cell nuclei stained in blue while α-SMA stained in red. Scale bar=100 µm. **(G)** Quantification of α-SMA staining area (%). ***p < 0.001. #*p* < 0.05, ##*p* < 0.01, ###*p* < 0.001 vs. PCL-HA/H-E group.

**Figure 8:**
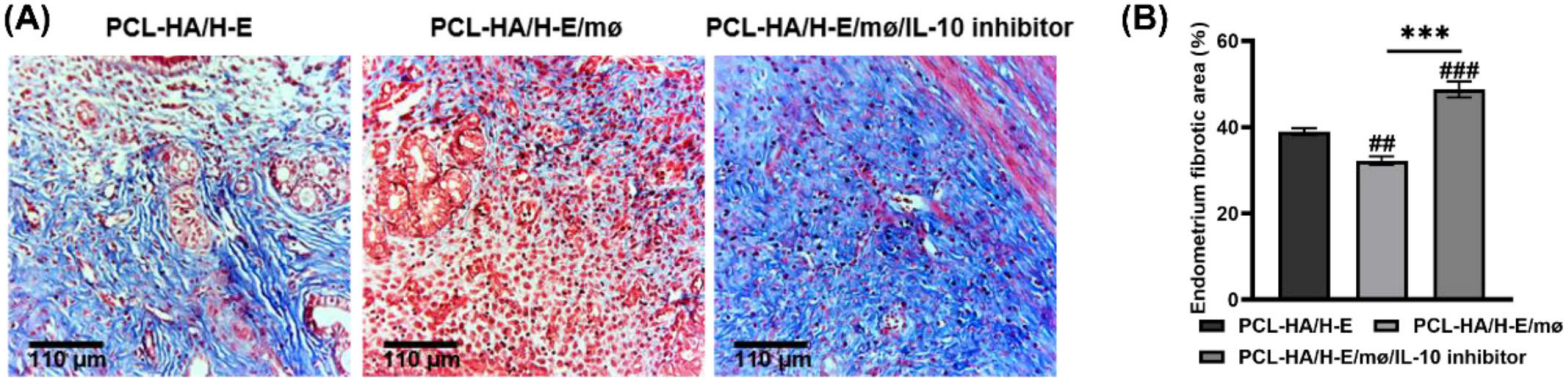
Masson staining of rat uterine tissues of the following groups: PCL-HA/H-E, PCL-HA/H-E/mø and PCL-HA/H-E/mø/IL-10 inhibitor, at day 7. **(A)** Representative Masson images of the rat uterine tissues of the different treatment groups at day 7. Scale bar=110 µm. **(B)** Quantification of the endometrium fibrotic area (%). ***p < 0.001. ##*p* < 0.01, ###*p* < 0.001 vs. PCL-HA/H-E group.

Previous studies have shown that IL-10 can inhibit skin, lung, kidney and cardiac tissue fibrosis by inhibiting fibrosis-associated miRNAs (miRNA-21, -145, and -208) and decreasing collagen I and III production (36–38). Based on the results obtained in sections 3.1-3.3, it appeared that the PCL-HA/H-E/mø co-delivery had enhanced both IL-10 gene and protein expression, while reducing the endometrial tissue fibrosis. As a result, further experiments were designed to investigate the relationship between the IL-10 gene and protein upregulation in the co-delivery treatment group and the decreased α-SMA expression and endometrial tissue fibrosis (**Figures 6**–**8**). As can be seen in **Figures 6A-E**, when the RFs cultured *in vitro* were treated with cell culture supernatants obtained from PCL-HA/H-E/mø co-seeding condition (7-day culture period), their IL-10 gene expression level was significantly increased when compared with the PCL-HA/H-E mono-seeding condition, or the control group. On the other side, the RFs treated with the co-seeding medium showed lower α-SMA gene expression vs. the mono-seeding or control groups (**Figure 6F**), which was the exact opposite of the IL-10 gene expression profile.

Since increased IL-10 gene expression was associated with decreased α-SMA expression, IL-10 inhibition studies were undertaken to test the hypothesis that IL-10 played significant role in down-regulating α-SMA expression and reducing endometrial tissue fibrosis. As can be seen in **Figure 6G**, when the RFs were treated with cell culture supernatant from the co-delivery condition plus the IL-10 inhibitor (PCL-HA/H-E/mø/IL-10 inhibitor), IL-10 gene expression of RFs was dramatically decreased to a level that was lower than the mono-delivery condition and the no inhibitor control. On the contrary, the RFs of the PCL-HA/H-E/mø/IL-10 inhibitor group showed increased α-SMA gene expression when compared to the no inhibitor and mono-delivery condition (**Figure 6H**).

For the *in vivo* IL-10 inhibition experiments, it was found that with the addition of the IL-10 inhibitor, the co-delivery treatment group showed lower IL-10 gene (**Figure 7A**) and protein expression (**Figures 7B–D**) in the endometrial tissues, when compared with the mono-delivery and co-delivery without IL-10 inhibitor groups, suggesting successful IL-10 inhibition in the *in vivo* endometrial damage model. The successful IL-10 inhibition in the endometrial damage model caused an increased α-SMA gene and protein expression, when compared with the co-delivery and mono-delivery conditions (**Figures 7E–G**).

Finally, Masson staining of rat uterine tissues (**Figure 8**) confirmed that adding IL-10 inhibitors in the co-delivery condition can interfere with the reduction of endometrial fibrosis. Hence, the IL-10 inhibition studies confirmed that the PCL-HA/H-E/mø co-delivery system exerted its effect on reducing endometrial tissue fibrosis (inhibiting the α-SMA expression) via the upregulation of the anti-inflammatory marker IL-10 expression.

## 4 Discussion

The endometrium, which consists of basal and functional layers, is a highly regenerative tissue that undergoes monthly growth, differentiation, and shedding during women’s reproductive age (39). Endometrial tissue injury and abnormal endometrial tissue repair are amongst the most important causes of female infertility (39). IUA is usually caused by curettage and iatrogenic injury, and its essence is endometrial fibrosis, which most often associates with persistent inflammation (40). At present, the different clinical interventions focus primarily on TCRA procedures or the injection of HA gel to treat IUA (1, 41). These interventions have had limited improvement on reproductive outcomes because the intrauterine devices cannot fundamentally prevent endometrial tissue fibrosis from re-occurring and often need to be removed, and HA gel has a short retention time in the uterus (41).

In recent years, different types of biomaterial scaffolds, pro-wound-healing/anti-fibrotic therapeutic factors, and MSC therapies have been investigated as more effective strategies to address IUA (42–44). Biomaterial nanofiber membranes constructed by electrospinning technology can have a three-dimensional structure mimicking natural ECM, with high surface area/volume ratio, good porosity, and strong mechanical properties, which can provide a suitable microenvironment for cell growth, proliferation, and the loading of bioactive factors (45, 46). The physical, chemical and biological properties of the nanofibrous electrospun scaffolds can be adjusted by changing the electrospinning parameters, for instance, the addition of natural ECM components can further improve the biocompatibility of the electrospun scaffolds with the local cells and ECM proteins (45). Additionally, it has been found that MSCs derived from various tissues, such as bone marrow, adipose tissue and placenta/umbilical cord, can migrate to areas of tissue injury, release anti-inflammatory cytokines and promote immunomodulation and wound healing (47, 48). Notably, bone marrow MSCs have been shown to promote M2-type polarization of mø phenotypes to enhance tissue repair and remodeling via direct cell-cell contact and the secretion of soluble factors (49). In a previous study, our group fabricated electrospun PCL-HA synthetic-natural composite membranes and they showed good structural and biochemical properties and exerted great potential to support H-EMSCs’ adhesion and proliferation (25). Additionally, H-EMSCs seeded on PCL-HA membranes showed self-renewal and low immunogenicity characteristics and could have the potential for endometrial tissue repair and regeneration (25).

In this study, considering the fact that both H-EMSCs and mø could play important roles in endogenous endometrial regeneration, we investigated the co-delivery of H-EMSCs and mø via PCL-HA membranes for treating endometrial fibrosis and IUA. It was observed that the PCL-HA electrospun membrane with H-EMSCs and mø co-seeding significantly reduced the endometrial fibrosis, enhanced M2 anti-inflammatory macrophage marker expression in the endometrial tissue injury model, when compared to the normal repair, PCL-HA membrane alone and PCL-HA with H-EMSCs mono-seeding groups. This current study suggested that the biocompatible PCL-HA electrospun membrane, when co-seeded with H-EMSCs and mø rather than just H-EMSCs, could have great potential in preventing endometrial fibrosis and promoting more natural endometrial tissue repair.

Using MSCs to educate mø to enhance tissue repair and regeneration has been reported previously in different tissues of the body. For instance, it was found that bone marrow derived-MSCs that were in direct contact with M1 pro-inflammatory decidual mø could promote their change to the M2 anti-inflammatory skew, by enhancing TNF-stimulated gene-6 (TSG-6) production and CD200 expression by the MSCs, in an abortion model (15). In addition, in a mouse IUA model, it was found that human umbilical MSCs pre-conditioned with IL-1β, TNF-α and IFN-γ promoted anti-inflammatory M2-type mø polarization, decreased endometrial tissue inflammation and fibrosis, and improved the immune microenvironment of endometrial regeneration by downregulating the Janus kinase/signal transducers and activators of transcription (JAK/STAT) signaling pathway (2). The interactions between MSCs and mø have not only attracted attention in the repair and regeneration of uterine tissues but also been investigated in sepsis and spinal cord injury models (50, 51). In the sepsis disease model, it was found that bone marrow MSCs produced prostaglandin E2 (PGE_2_), which bound to EP2 and EP4 receptors on the mø’s surfaces to induce anti-inflammatory cytokine IL-10 expression, to relieve sepsis symptoms (52). Additionally, bone marrow MSCs enhanced the polarization of M2-type mø phenotypes in a spinal cord injury model and promoted effective spinal tissue repair, indicating that the bone marrow MSCs were critical for balancing the M1/M2 polarization states of the mø for normal spinal tissue repair (51). More interestingly, it was also found that bone marrow MSCs can promote M2 mø polarization by changing the metabolic status through a PGE_2_-dependent mechanism (53).

The observations of the current study agreed well with those previous findings, demonstrating that the endometrial co-delivery system, fabricated by co-seeding H-EMSCs and mø onto PCL-HA composite electrospun membranes, promoted the overall change of a more M1-type to a more M2-type mø phenotypes, upregulated anti-inflammatory cytokine IL-10 expression, downregulated pro-inflammatory factor TNF-α expression. What is particularly important and a novel finding for the literature here, is that at the dose and ratio of cells used for delivering the two cell types on a foreign biomaterial, a pro-inflammatory to anti-inflammatory endometrial tissue microenvironment change was achieved in a manner that appeared to have contributed to the inhibition of endometrial fibrosis rather than yielding a classical foreign body fibrotic response. The presence of the biomaterial enabled the co-delivery which appeared to be important since the co-delivery patch induced more CD90 gene and protein expression in the damaged endometrial tissue vs. other conditions, including that of the H-EMSCs alone. Such findings indicated that when mø were co-delivered in proximity with the H-EMSCs via PCL-HA, the retention of the seeded H-EMSCs or recruitment of the H-EMSCs reduced fibrosis and enabled enhanced repair of the native endometrial tissue. This aligned well with the roles that M2 type mø play, in promoting tissue repair by MSCs (13, 54). However, it should be noted that macrophage polarization *in vivo* is more dynamic than a strict M1/M2 dichotomy (55). Specifically, macrophages are only broadly categorized into two primary functional types: classically activated (M1) macrophages and alternatively activated (M2) macrophages. The M1 type is proinflammatory, specialized in pathogen and tumor clearance through the robust production of pro-inflammatory cytokines (56). The M2 type encompasses several subtypes, including the wound-healing (M2a) and regulatory (M2b, M2c) macrophages, which are involved in tissue repair and immune modulation (56). However, studies have revealed a significant overlap *in vivo*, where macrophages within the same tissue microenvironment can co-express markers characteristic of both M1 and M2 states (55, 57). Therefore, macrophages are best understood not as rigid, discrete populations, but as a plastic continuum of functional states.

It was of critical importance in this work to show the nature of the anti-fibrotic pathway associated with the delivery vehicle and its payload of cells, as PCL is well known to induce chronic foreign body responses (58), which would negate the outcomes that the authors were seeking to overcome. In fact, the PCL-HA controls in this work showed greater fibrosis when not carrying the co-seeded cells. However, in this particular processed form and combination of the co-seeded PCL-HA patch, the authors observed a significantly reduced fibrosis in the endometrial tissue damaged model. HA is a natural linear anionic polysaccharide derived from native ECM, and has good hydrophilicity, biocompatibility and non-immunoreactivity (24). Previous studies showed that HA can modulate skin tissue inflammation and wound-healing by binding to fibrinogen to activate the clotting pathways, inhibiting neutrophil migration to decrease inflammation, and stimulating the secretion of matrix metalloproteinases for promoting angiogenesis (59–61). Our previous study also revealed that H-EMSCs cultured on PCL-HA showed decreased IL-6 gene expression and increased IL-10, VEGFA, TGF-β gene expression vs. PCL-SF and PCL (25). The unique physiochemical and biological characteristics of this specific PCL-HA formulation might have contributed to the attenuated fibrosis observed in this study.

According to the literature, IL-10 has oftentimes been considered as an anti-inflammatory cytokine that has anti-fibrotic properties in tissue repair and remodeling (62). Consequently, the study investigated whether more IL-10 production in the co-delivery condition could be contributing to the reduced endometrial fibrosis. With successful blocking of IL-10 in the *in vitro* and *in vivo* experiments, it was found that the fibrotic marker α-SMA expression was significantly increased at both the gene and protein levels. Additionally, the Masson staining of the endometrial tissues for the IL-10 inhibitor group also showed dense collagenous fibrotic tissue formation. These findings provided substantial support that a key mechanistic pathway associated with the anti-fibrotic effects of PCL-HA based co-delivery of H-EMSCs and mø, was related partially to IL-10 released in the local endometrial tissue micro-environment. This finding agreed well with anti-fibrotic studies in the repairing of other tissue types. For example, viral vector-mediated IL-10 gene transfer into skin wounds led to regenerative healing, and the ECM formed was both morphologically and biomechanically indifferent when compared with that of the unwounded skin tissue (63). In addition, it was also found that the application of IL-10 in a myocardial infarction model suppressed pro-inflammatory cytokine production, inhibited inflammatory cell infiltration and reduced myocardial tissue fibrosis (64). Similarly, it is noted in the literature that a deficiency in IL-10 could aggravate kidney inflammation and fibrosis in a unilateral ureteral obstruction mouse model (65). Although IL-10 is implicated in the anti-fibrotic effect of the co-delivery system, the cellular source of IL-10 *in vivo* remains unclear (macrophages vs. H-EMSCs vs. other cells), future studies will identify the cellular source of IL-10 *in vivo* by combining genetic tools with advanced immunofluorescence-based cytokine tracing techniques. Additionally, fibrosis evaluation in this study is based on Masson staining and α-SMA expression, next-step studies could further examine the expression of collagen-specific markers in the different conditions to compare with the Masson staining and α-SMA expression data.

Further, it should however be noted that IL-10 likely is not alone responsible for the enhanced repair and may only be one of the factors that the co-delivery was able to activate towards the inhibition of the endometrial fibrosis due to the close proximity of the cells, which enabled effective chemokine communication. Future studies will be investigating other prominent signaling molecules and pathways (e.g., IGFBP3, IL-17A, RhoA/ROCK1/MYL9 (66–68)) involved in down-regulating endometrial fibrosis, using the PCL-HA based H-EMSCs-mø co-delivery system.

## 5 Conclusions

In summary, this study established a rat endometrial damage model and probed the application of co-delivery of H-EMSCs and mø, via a PCL-HA patch-based system. It was found that the co-delivery system could significantly reduce fibrosis in the endometrial tissue damage model. The co-delivery patch supported an overall M1-type to M2-type change of the mø phenotypes and increased MSC numbers in the endometrial tissue. It appeared that IL-10 played an important role in reducing endometrial fibrosis, mediated by the H-EMSCs and mø co-seeded PCL-HA patch. The study provides significant insights into using an H-EMSCs-mø co-delivery system for effectively alleviating endometrial fibrosis and IUA in the future.

## Data Availability

The original contributions presented in the study are included in the article/**Supplementary Material**. Further inquiries can be directed to the corresponding authors.

## Supplementary material

Supplementary Figure 1 Rat endometrial injury model and implantation of PCL-HA based endometrial patches. **(A–C)** After anesthesia, the rat abdomen was dissected to expose the uterine horn. **(D, E)** Creating the endometrial lesion in the rat uterus, with a sterile needle. **(F–H)** Implanting the PCL-HA based endometrial patches onto the endometrial lesion area. **(J)** Closing the wound with absorbable sutures.
