# Supplementary figures and images for: Co-delivery of endometrial mesenchymal stem cells and macrophages with an electrospun patch suppresses endometrial fibrosis via IL-10 related signaling
Source: Front Immunol. 2026 Feb 19;17:1750456. doi: 10.3389/fimmu.2026.1750456 (PMC12960166; doi:10.3389/fimmu.2026.1750456)

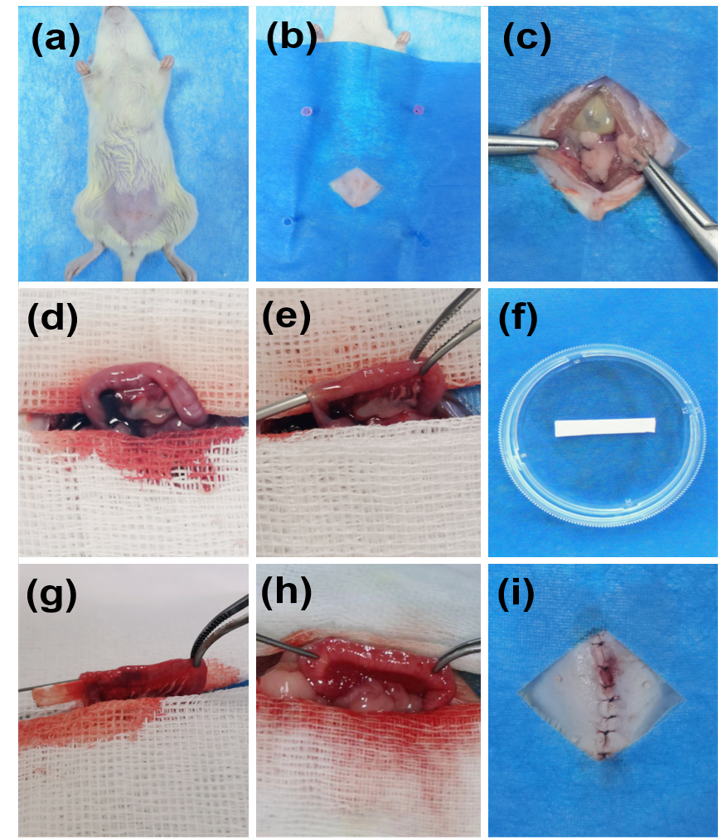

Supplement: Supplementary Figure 1 — Rat endometrial injury model and implantation of PCL-HA based endometrial patches. (A–C) After anesthesia, the rat abdomen was dissected to expose the uterine horn. (D, E) Creating the endometrial lesion in the rat uterus, with a sterile needle. (F–H) Implanting the PCL-HA based endometrial patches onto the endometrial lesion area. (J) Closing the wound with absorbable sutures. [file Image1.tif]
