# Supplementary material for: Co-delivery of endometrial mesenchymal stem cells and macrophages with an electrospun patch suppresses endometrial fibrosis via IL-10 related signaling
Source: Front Immunol. 2026 Feb 19;17:1750456. doi: 10.3389/fimmu.2026.1750456 (PMC12960166; doi:10.3389/fimmu.2026.1750456)
Supplement: Supplementary file 2 [file Table1.docx]

**Supplemental Table 1**. Clinical information of patients who donated endometrial tissue samples of the study.

| **Patient No.** | **Age (years)** | **BMI** | **Health condition** | **Menstrual cycle stage** | **Collected tissue type** |
| --- | --- | --- | --- | --- | --- |
| **1** | 23 | 18.9 | healthy | menstrual cycle day 2-4 | endometrial tissue pieces |
| **2** | 35 | 22.1 | healthy | menstrual cycle day 2-4 | endometrial tissue pieces |
| **3** | 28 | 23.9 | healthy | menstrual cycle day 2-4 | endometrial tissue pieces |
| **4** | 26 | 20.6 | healthy | menstrual cycle day 2-4 | endometrial tissue pieces |
| **5** | 26 | 24.2 | healthy | menstrual cycle day 2-4 | endometrial tissue pieces |
| **6** | 29 | 20.9 | healthy | menstrual cycle day 2-4 | endometrial tissue pieces |
